# The impact of cut-soiler technology on rice-wheat production in salt-affected areas of western Indo-Gangetic Plains of India

**DOI:** 10.1371/journal.pone.0320775

**Published:** 2025-04-01

**Authors:** Guenwoo Lee, Junya Onishi, Taishin Kameoka, Kayo Matsui, Gajender Yadav, Suresh Kumar, Govind Prasad, Satender Kumar, Rajender Kumar Yadav

**Affiliations:** 1 Social Sciences Division, Japan International Research Center for Agricultural Sciences (JIRCAS), Tsukuba, Ibaraki, Japan,; 2 Rural Development Division, Japan International Research Center for Agricultural Sciences (JIRCAS), Tsukuba, Ibaraki, Japan,; 3 Division of Soil and Crop Management, ICAR–Central Soil Salinity Research Institute, Karnal, Haryana, India,; 4 Division of Social Science Research, ICAR–Central Soil Salinity Research Institute, Karnal, Haryana, India,; 5 ICAR–Central Soil Salinity Research Institute, Karnal, Haryana, India; ICAR - IIFSR: ICAR - Indian Institute of Farming Systems Research, INDIA

## Abstract

Soil salinization poses a significant challenge to agricultural productivity worldwide, particularly in the rice-wheat belt of the western Indo-Gangetic Plains (IGP), where excess sodium salts (sodicity) degrade soil health and threaten crop production. The cut-soiler, a farm machine developed in Japan, is an innovative and cost-effective solution. The cut-soiler constructs residue-filled, shallow subsurface drains, enhancing lateral and vertical water movement through the soil and improving soil conditions and crop productivity. Unlike previous studies confined to semi-controlled experimental settings, this research uniquely evaluates the effectiveness of cut-soiler technology on local farms severely affected by salinization, specifically addressing subsurface sodicity and recurrent waterlogging conditions that hinder agricultural profitability. From 2019 to 2023, feasibility trials were conducted in farmers’ fields across Punjab and Haryana, India, where gypsum was applied alongside crop residue to enhance subsurface soil reclamation. The analysis reveals significant improvements in the rice and wheat yields. The findings suggest that the application of a cut-soiler over an area of 20 hectares produces a positive net present value (NPV > 0), a benefit-cost ratio (BCR > 1), and an internal rate of return (IRR > 10%), thus supporting the financial viability of the investment for the reclamation of sodicity-affected regions. In addition, the cut-soiler reduces crop residue burning, contributing to environmental sustainability. The substantial yield increases observed in both experimental and conventional farming settings highlight the potential of cut-soiler technology to transform agricultural practices in salt-affected regions, improve crop productivity, and boost economic returns for local farmers.

## Introduction

Soil alkalinity, characterized by high pH levels (typically above 8.5) and excess sodium ions (sodicity), significantly challenges agricultural productivity worldwide. Sodicity adversely affects soil structure and nutrient availability, resulting in poor soil health. According to recent Food and Agriculture Organization (FAO) estimates, the total area of salt-affected soils in the world is 1, 381 million hectares, including saline, saline-sodic, and sodic soils, or 10.7 percent of the total global land area [1]. Natural processes and unsustainable agricultural practices, including irrigation with high-sodium water and insufficient drainage systems, further exacerbate this issue.

In India, sodic soils represent a substantial barrier to sustainable agriculture, particularly in Haryana and Punjab, where groundwater—a primary irrigation source—often contains high Residual Sodium Carbonate (RSC) levels, contributing to increased soil sodicity [2]. While India has reclaimed approximately 2.8 million hectares of sodic soils, primarily in the Indo-Gangetic Plains (IGP) [3], these efforts are largely confined to the surface layer. Subsurface sodicity remains a pressing concern, exacerbated by poor drainage, high evaporation, and inadequate leaching, which force sodium ions deeper into the soil profile. Existing reclamation methods, such as gypsum application, often fail to address subsurface issues, risking re-sodification when sodium ions are not adequately leached from the root zone [4]. The high clay content and poor permeability of sodic soils further contribute to seasonal waterlogging, especially during the monsoon season, further hindering agricultural productivity.

Addressing subsurface sodicity, waterlogging, and extending reclamation to the entire root zone requires innovative soil management practices. However, existing methods for the targeted application of soil amendments, such as gypsum and crop residues, to subsurface layers are neither economically nor technically feasible [5]. This underscores the urgent need for cost-effective, scalable, and sustainable solutions tailored to local farming contexts.

The cut-soiler, developed in Japan, emerges as a promising innovation for managing subsurface sodicity. Originally designed to improve drainage efficiency in Japanese paddy fields [6], the cut-soiler offers a cost-effective method for targeted placement of crop residues and soil amendments at subsurface depths. By creating shallow subsurface drains enriched with approximately 5–6 tons of rice residue per hectare, the cut-soiler addresses subsurface sodicity while repurposing crop residues that are otherwise burned. This dual functionality of cut-soiler not only mitigates air pollution but also aligns with the Government of India’s Land Degradation Neutrality (LDN) targets and multiple Sustainable Development Goals (SDGs), including Goal 12 (Responsible Consumption and Production), Goal 13 (Climate Action), and Goal 15 (Life on Land).

Preliminary studies in controlled experimental settings have demonstrated the cut-soiler’s potential to improve drainage, reduce subsurface sodicity, and enhance crop yields in salt-affected areas [4,7]. However, limited evidence exists on its effectiveness in farmers’ fields wherein other external factors such as socio-economic conditions, farmers’ managerial capacity, varying field features, and input-use pattern (heterogeneous conditions) may affect the intended outcome of the application of the cut-soiler. Therefore, the effect of cut-soiler in real-field settings is needed for generating empirical, robust, and credible evidence on its crop productivity-enhancing potential in salt-affected regimes. Recognizing the importance of bridging this knowledge gap, the present study revalidates the cut-soiler’s impact under field conditions using data from farms managed by local farmers in Haryana and Punjab.

From 2019 to 2023, this study evaluated the cut-soiler’s performance across 64 plots managed by 38 farmers in these states. Employing robust statistical methods, including difference-in-differences (DID), heterogeneous DID, and analysis of covariance (ANCOVA), the study quantified the cut-soiler’s impact on rice and wheat yields. The results reaffirm the technology’s efficacy in improving agricultural productivity under challenging salt-affected conditions. Additionally, the technology’s ability to reduce crop residue burning highlights its environmental benefits.

However, despite these advantages, several economic barriers hinder the widespread adoption of this technology. For example, the annual fixed cost of a cut-soiler is estimated at 480,255 INR, while the annual savings in a rice-wheat system amount to just 34,262 INR and 8,750 INR per hectare, respectively. Break-even analysis shows that managing at least 18.82 hectares is required to cover these fixed costs. However, given that the average farm sizes in Haryana (2.25 hectares) and Punjab (3.66 hectares) fall considerably short of the required threshold.

Several strategies are recommended to address these economic barriers and encourage widespread use. These include providing government subsidies, implementing optimized leasing systems, enabling collective purchasing, and producing the equipment in low-wage countries. By lowering adoption costs, these approaches can unlock the full potential of cut-soilers, delivering both economic and environmental benefits on a broader scale.

The remainder of this paper is organized as follows: The cut-soiler for soil salinity management section details the mechanism of targeted residue and gypsum placement using the cut-soiler. The materials and methods section presents the design of the field experiments. The empirical analyses section outlines the estimation methods, and the results section presents the results. The discussion section explores strategies for widespread adoption, and the conclusion section concludes with policy implications and future research directions.

### Cut-soiler for soil salinity management

**Fig 1:**
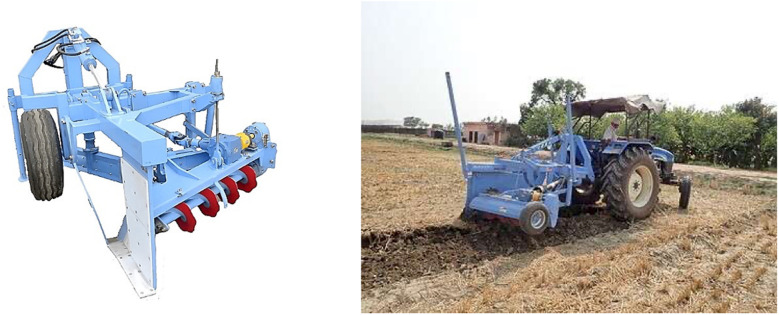
Outlook of Cut-soiler.

The cut-soiler (KKSR-02), developed by the National Agriculture and Food Research Organization (NARO) in Japan, is an affordable technology designed to enhance drainage as part of standard farming operations. As depicted in Fig 1, this tractor-mounted attachment utilizes surface crop residues to construct shallow subsurface drainage channels. The unit comprises a blade and a robust frame, which measures 2,000 mm in length, 1,500 mm in width, and 1,650 mm in height and weighs 700 kg. It is manufactured and sold by Hokkai Koki Co., Ltd. in Japan [8].

“Reprinted from [8] under a CC BY 4.0 license. Copyright © 2023 by JIRCAS. Used with permission. The original figure is available at https://www.jircas.go.jp/sites/default/files/publication/manual_guideline/manual_guideline-jircas-2022-001_-.pdf (see page 21)”

The cut-soiler offers a cost-effective option considering the heavy machinery and manpower required for other available sub-surface reclamation and drainage techniques. When deployed by individual farmers or cooperatives, the cut-soiler can significantly lower the costs of root zone reclamation and improve drainage over extensive areas, making it an economically viable option.

**Fig 2:**
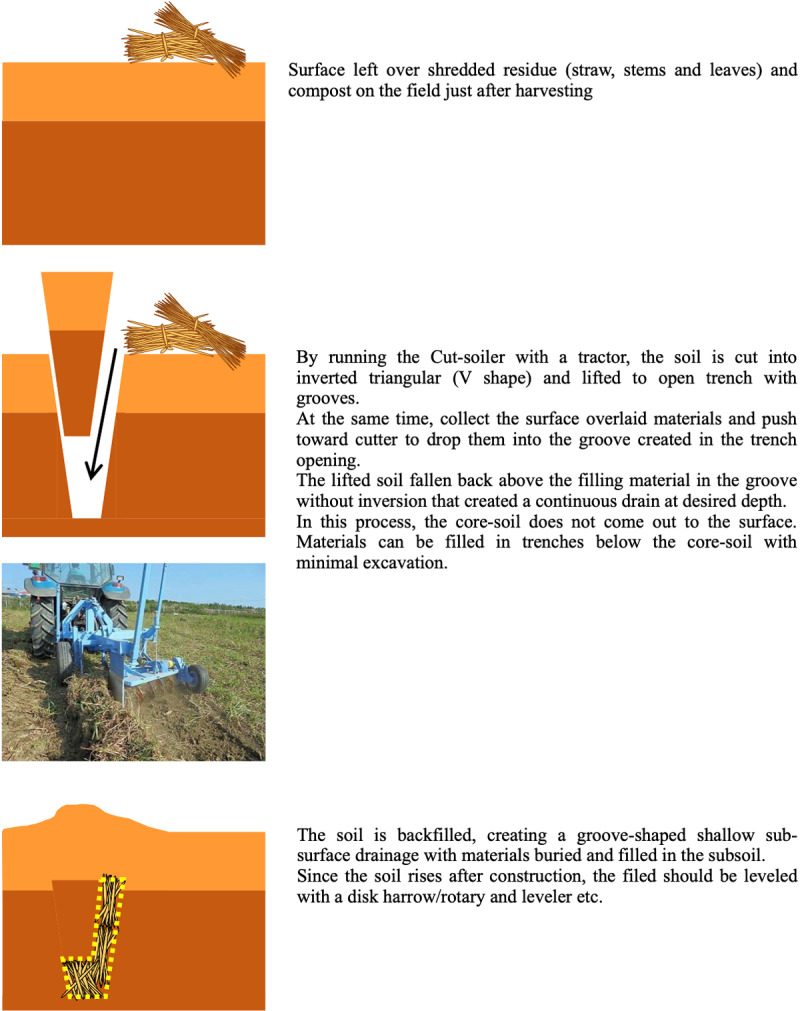
Mechanism of Sub-surface Drainage Construction with the Cut-soiler.

Fig 2 illustrates the subsurface drainage construction process facilitated by the cut-soiler machine. The machine cuts into the soil to create a V-shaped furrow and lifts the excavated soil. It then strategically places scattered surface materials such as straw/residue and/or gypsum at the bottom of the furrow. Finally, the lifted soil is overlaid onto these materials to complete the subsurface drainage channel effectively. For a more detailed explanation of this process, refer to Fig 2.

“Reprinted from [8] under a CC BY 4.0 license. Copyright © 2023 by JIRCAS. Used with permission. The original figure is available at https://www.jircas.go.jp/sites/default/files/publication/manual_guideline/manual_guideline-jircas-2022-001_-.pdf (see page 23)”

The cut-soiler provides a comprehensive pathway to enhance agricultural productivity and sustainability, particularly in salt-affected regions. The process begins with embedding crop residues and soil amendments, such as gypsum, into the soil during the construction of shallow subsurface drainage channels. This critical intervention reduces salt accumulation by facilitating leaching and enhancing water infiltration, creating an optimized root-zone environment conducive to healthy crop growth. At the next stage, improved soil structure and nutrient use efficiency result from integrating organic matter and amendments, which help stabilize soil aggregates and enhance microbial activity. These improvements promote better root development, increasing nutrient uptake and higher crop productivity. Over time, incorporating organic residues enriches soil organic matter, fostering long-term improvements in soil fertility and resilience to environmental stress.

Empirical evidence supports the efficacy of this approach Neha, Yadav (8) found that shallow sub-surface drainage built with the cut-soiler at a depth of 60 cm reduced soil salinity (measured as the electrical conductivity of the solution extracted from saturated soil–ECe:) by 8% (not statistically significant) and 32% (P=0.047) at 4 and 16 months after construction, respectively. Additionally, the study highlights yield increases of 4% (not statistically significant) for dry-season crops (mustard from November to March) and 23% (P=0.048) for rainy-season crops (pearl millet from June to September). Furthermore, several studies [4,9,10] have shown that the use of gypsum and rice residue, particularly when facilitated by a cut-soiler, effectively reduces subsurface sodicity and improves crop yields in the IGP’s sodic soils.

However, as these findings have not yet been validated through experiments on local farms, there remains room for further research to determine if the cut-soiler can have a positive effect on yield increases in general farms managed by local farmers in salt-affected areas, as observed in controlled experimental farms by researchers.

## Materials and methods

The primary objective of this study is to evaluate the direct impact of cut-soiler intervention on agricultural productivity under real-world conditions in farmer-managed fields. The cut-soiler was employed to construct residue-filled, shallow subsurface drains on selected plots, with contractors specifically hired to conduct the construction. Farmers were informed about the anticipated productivity improvements associated with the technology. Hands-on training and demonstration of operating the cut-soiler were provided to the farmers under various outreach and extension programs of ICAR-Central Soil Salinity Research Institute, Karnal, India. This design ensures that the observed changes in productivity can be attributed primarily to the direct effects of the intervention itself, minimizing the impact of potential confounding variables.

To this end, we conducted an extensive plot-level survey from 2019 to 2023, collecting data from 22 plots in Haryana and 42 plots in Punjab managed by 38 local farmers. Haryana and Punjab are recognized as the two most agriculturally productive states of the western IGP, often referred to as India’s breadbasket. Specifically, Punjab is known for its substantial wheat and rice yields, making it a major contributor to India’s grain supply. Haryana is a prominent producer of staple crops [11]. However, both states face soil salinity and/or sodicity challenges, which significantly threatens their agricultural productivity and sustainability [3].

**Fig 3:**
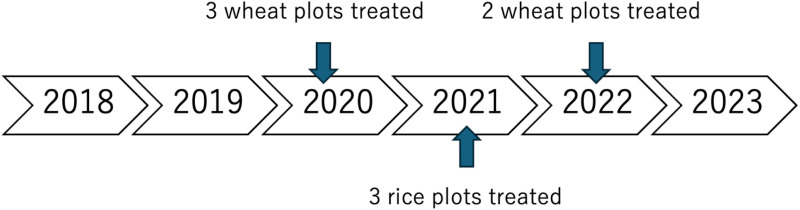
Treatment Timeline.

Table 1 shows that of the 64 plots surveyed, cut-soiler drains were constructed in three rice plots and five wheat plots. Purposive and random sampling was employed to select farmers for the study. Karnal and Kaithal districts in Haryana and Patiala district in Punjab (India) were purposively selected due to the prevalence of issues such as sodicity, sub-surface sodicity, and the application of irrigation with saline water. Highly affected patches were identified within these districts, and a list of farmers was compiled from these areas. From this list, 38 farmers were selected who were willing to implement the cut-soiler technology on their plots, totaling 64 plots. Due to limited resources (only one cut-soiler machine), the technology was implemented on eight plots over 5 years to demonstrate its technical feasibility and effectiveness in achieving the intended outcomes. The remaining plots, located near the treated plots, were designated as the control group to minimize or avoid the influence of other climatic and soil-related factors. This selection method ensures that the technology is applied where it is most needed; however, it may limit the external validity and generalizability of the findings, as farmers with higher motivation or better access to resources are more likely to be included in the study. The operational schedule for the cut-soiler treatments, which provides a detailed overview of the timing and frequency of the interventions, is shown in Fig 3.

**Table 1:** Balance test on pre-treatment data.

		Total	Non-treated	Treated	Diff.
**Rice**	**Household-level characteristics**				
	HH’s gender	1.00	1.00	1.00	0.00
	HH’s age	54.00	53.57	62.67	-9.09
	HH’s education level	2.00	2.00	2.00	0.00
	No. family workers on farm	1.72	1.75	1.00	0.75^*^
	**Plot-level characteristics**				
	Plot size (acre)	3.94	4.07	1.33	2.73
	Tube well irrigation (1=yes)	0.94	0.93	1.00	-0.07
	HH’s perception of salinity	3.47	3.43	4.33	-0.91
	UREA applied (kg/acre)	2.96	2.96	3.00	-0.04
	DAP applied (kg/acre)	0.03	0.03	0.00	0.03
	ZINC applied (kg/acre)	0.93	0.93	1.00	-0.07
	MOP applied (kg/acre)	0.063	0.07	0.00	0.07
	Organic fertilizer applied (kg/acre)	15.58	16.34	0.00	16.34
	Gypsum applied (1=yes)	0.16	0.15	0.33	-0.19
	Yield (100kg/acre)	13.67	13.86	9.86	4.01
	*N*	64	61	3	
**Wheat**	**Household-level characteristics**				
	HH’s gender	1.00	1.00	1.00	0.00
	HH’s age	54.00	54.03	53.60	0.43
	HH’s education level	2.00	1.98	2.30	-0.22
	No. family workers on farm	1.92	1.95	1.60	0.35
	**Plot-level characteristics**				
	Plot size (acre)	3.89	4.12	1.20	2.92
	Tube well irrigation (1=Yes)	0.94	0.93	1.00	-0.07
	HH’s perception of salinity	3.47	3.41	4.20	-0.79
	UREA applied (kg/acre)	2.98	2.98	3.00	-0.02
	DAP applied (kg/acre)	0.00	0.00	0.00	0.00
	ZINC applied (kg/acre)	0.11	0.10	0.20	-0.10
	MOP applied (kg/acre)	0.00	0.00	0.00	0.00
	Organic fertilizer applied (kg/acre)	100.47	89.15	234.00	-144.85^*^
	Gypsum applied (1=yes)	0.09	0.08	0.20	-0.12
	Yield (100kg/acre)	12.90	13.03	11.40	1.63
	*N*	64	59	5	

Note: HH, Head of Household. The scale for HH’s perception of plot salinity ranges from 1 (optimal) to 6 (poor).

***p<0.01,

**p<0.05,

*p<0.1.

Table 1 also presents the balance test results comparing the treated and untreated groups. This test is crucial for verifying whether the treatment and control groups are comparable in terms of key characteristics, which is essential for validating the findings of our study. For rice plots, no significant differences are observed in most household- and plot-level characteristics between the treatment and control groups, except for the number of family workers on the farm, where treated plots have significantly fewer family workers (0.75, p<0.1). Similarly, for wheat plots, no significant differences are found in most variables, apart from organic fertilizer application, where treated plots applied significantly more organic fertilizer (234 kg/acre) compared to untreated plots (89.15 kg/acre, p<0.1). These results suggest that the treatment and control plots are reasonably well-matched regarding critical attributes within the study context, minimizing the likelihood of systematic differences. However, the observed differences in family labor for rice plots and organic fertilizer application for wheat plots could influence the outcomes and be interpreted cautiously. Furthermore, differences in baseline characteristics and farming practices across Haryana and Punjab’s diverse agricultural zones may impact the scalability of these findings.

## Empirical analyses

### Methodologies for evaluating the treatment effects of cut-soiler

**Fig 4:**
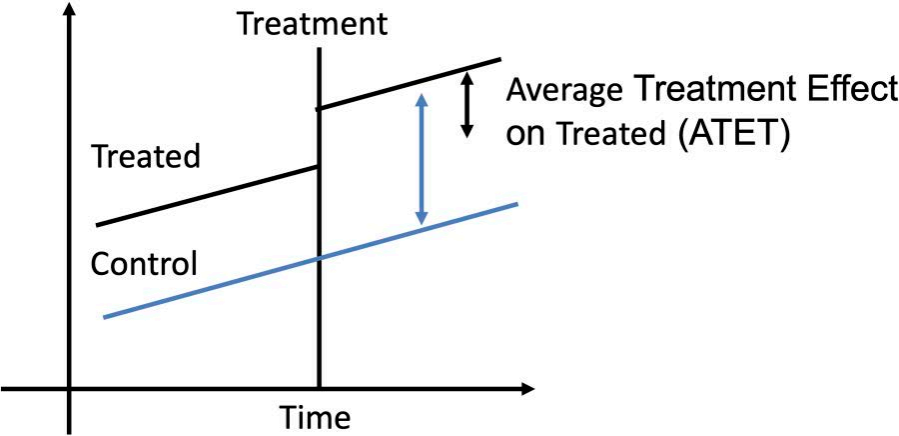
Difference-in-differences (DID).

In this study, the DID approach is utilized to evaluate the differences in yield and revenue between plots treated with cut-soilers and untreated plots, which serve as the control group. This method goes beyond a simple comparison by examining changes in yields over time within and between these groups, enabling the isolation of the causal impact of the cut-soiler treatment. The DID approach is particularly valuable as it accounts for unobserved time-varying factors and differences in baseline trends that could influence outcomes. By leveraging the interaction between treatment timing and group status, this robust statistical framework ensures that the estimated effects are attributable to the cut-soiler intervention rather than to external factors or random variations. As illustrated in Fig 4, the DID method measures outcome changes before and after treatment, enabling a comprehensive analysis of treatment effects.

Using this approach, the pure treatment effect, known as the Average Treatment Effect on the Treated (ATET), can be effectively isolated. This result is achieved by accounting for and removing the influence of other confounding factors that may have occurred concurrently and may have influenced the outcome variable [12]. Such a rigorous methodological framework ensures that the observed differences in yield and revenue can be more confidently attributed to the treatment rather than to external variables. The proposed model reads as follows:


$$Y_{i,t}=\alpha +\beta_{1}Post_{t}+\beta_{2}Treat_{i}+\beta_{3}\left(Post_{t}Treat_{i}\right)+\varepsilon_{i,t},$$ (1)

where $Y_{it}$ represents the yield or revenue of the *i*^th^ plot at time *t*, *α* is the intercept term representing the baseline average level of the outcomes, $Post_{t}$ is a binary variable that equals 1 for the post-treatment period and 0 for the pre-treatment period, and $Treat_{i}$ is a binary variable that equals 1 if plot *i* is in the treated group and 0 otherwise. $Post_{t}Treat_{i}$ is an interaction term between the treatment group and the post-treatment period, $\varepsilon_{it}$ is the error term ($\varepsilon_{it}\sim iidN\left(0,\sigma^{2}\right)$, $\beta_{1}$ captures the difference between the treatment and control groups before the treatment or captures any secular trend in the outcome over time in the control group, $\beta_{2}$ captures the difference between the pre- and post-treatment periods for the control group, and $\beta_{3}$, the interaction term, captures the effect of the treatment, also known as ATET or DID estimate.

Including a vector of control variables in the DID model enhances the precision and validity of the estimated treatment effect ($\beta_{3})$ [12]. Therefore, we test a model that controls for variable *X’*, encompassing both household-level characteristics—such as the household head’s gender, age, education level, and the number of family members engaged in farming—and plot-level characteristics, including the plot size, use of tube well irrigation, use of gypsum, household head’s perception of salinity, and application volumes of inputs such as UREA, DAP, ZINC, MOP, and organic fertilizers. The model reads as follows:


$$Y_{i,t}=\alpha +\beta_{1}Post_{t}+\beta_{2}Treat_{i}+\beta_{3}\left(Post_{t}Treat_{i}\right)+X^{\prime }\gamma +\varepsilon_{i,t}.$$ (2)

In addition, given that the cut-soiler treatment was applied to wheat plots in 2020 and 2022, we utilize a heterogeneous DID approach. This advanced method enables us to systematically assess and quantify the variable treatment effects arising from the staggered implementation times, thereby offering a more detailed analysis of the impact of the intervention. The proposed model reads as follows:


$$\begin{array}{l} Y_{i,t}=\alpha +\beta_{1}Post2020_{t}+\beta_{2}Post2022_{t}+\beta_{3}Treat_{i}\\ +\beta_{4}\left(Post2020_{t}Treat_{i}\right)+\beta_{5}\left(Post2022_{t}Treat_{i}\right)+X^{\prime }\gamma +\varepsilon_{i,t}, \end{array}$$ (3)

where $Post2020_{t}$ and $Post2022_{t}$ are binary variables equal to 1 for the periods after the 2020 and 2022 treatments, respectively, and 0 otherwise, while $\beta_{4}$ and $\beta_{5}$ capture the effect of the 2020 and 2022 treatments on the treated plots relative to the untreated plots, respectively.

According to McKenzie [13] when autocorrelations are low, using the ANCOVA instead of the DID approach can substantially improve statistical power. Given the likelihood of low autocorrelations among the variables in our collected data, we employ an ANCOVA as a robustness check in our analyses, as illustrated in the following model:


$$Y_{i,t}=\alpha +\beta_{1}Treat_{i,t}+\beta_{2}Treat_{t}+\beta_{3}Y_{i,pre}+X^{\prime }\gamma +\theta_{t}+\varepsilon_{i,t},$$ (4)

where $Y_{i,pre}$ is the pre-treatment outcome variable for plot *I,* and $\theta_{t}$ represents time fixed effects.

### Financial analysis of cut-soiler technology

As mentioned above, the cut-soiler technology requires huge amount of the upfront investment. Its financial viability requires its large-scale adoption. To this end, we attempted to quantify the break-even (BE) points in terms of the minimum area to be covered under cut-soiler. First, the cost of the cut-soiler (fixed cost) was annualized using the following formula:


$$Annulaizedcostofcutsoiler=\frac{costofcutsoiler*r}{1-{\left(1+r\right)}^{-n}}$$ (5)

where, *r* is discount rate, that is, 10%, and n=expected life of cut-soiler, that is, 15 years. Second, the break-even coverage was estimated using the formula given below:


$$BE\left(ha\right)=\frac{fixedcostperannum\left(INR\right)}{Return\left(INRperha\right)}$$ (6)

where, return (INR per hectare) is the difference of saving (INR per hectare) and variable cost (INR per hectare).

Further, other parameters of financial viability (i.e., net present value [NPV]), benefit-cost ratio (BCR), and internal rate of return (IRR) were estimated assuming the different scenarios, that is, 20, 25, 30, 35, and 40 hectares of rice-wheat system under cut-soiler per year.

## Results

### Impact of cut-soiler treatment on the yield and revenue of rice and wheat: Difference-in-differences and heterogeneous difference-in-differences

Table 2 illustrates the effects of the cut-soiler treatment on rice and wheat yields through DID analysis. For rice, the results without control variables indicate a yield increase of 2.12 quintals per acre (0.52 tons per hectare), statistically significant at the 10% level. Incorporating control variables slightly reduces this increase to 1.38 quintals per acre (0.34 tons per hectare), which is no longer statistically significant. Similarly, the revenue impact for rice shows a decrease of 3,253.53 INR per acre (8,041.46 INR per hectare) without controls and 5,145.76 INR per acre (12,711.29 INR per hectare) with controls; neither is statistically significant. These results suggest that while there is evidence of yield improvements, their robustness weakens after adjusting for household- and plot-level covariates.

**Table 2:** Results from the DID analysis: Rice and wheat cultivation.

	Rice				Wheat			
	Yield (100kg/acre)		Revenue (INR/acre)		Yield (100kg/acre)		Revenue (INR/acre)	
	without X	with X	without X	with X	without X	with X	without X	with X
ATET	2.12^*^	1.38	-3253.53	-5145.76	2.54^**^	2.83^**^	5150.18^***^	5712.28^**^
	(2.5)	(1.29)	(-0.40)	(-0.73)	(5.45)	(4.70)	(5.64)	(4.87)
*N*	320	320	320	320	320	320	320	320
Cluster	64	64	64	64	64	64	64	64

Note: Average Treatment Effect on the Treated (ATET) estimate adjusted for panel effects and time effects. In addition to the variables in the table above, the models are adjusted for a range of covariates (X), including household-level variables such as the gender, age, and education level of the household head, along with the number of family members engaged in farm labor. Additionally, plot-level characteristics are accounted for, including plot size, the use of tube well irrigation, the use of gypsum, the household head’s perception of salinity, and the application volumes of inputs such as UREA, DAP, ZINC, MOP, and organic fertilizers. The analysis employs Wild bootstrap resampling with 1,000 iterations, and T-values are reported in parentheses.

***p<0.01,

**p<0.05,

*p<0.1.

For wheat, the DID analysis reveals more consistent and significant effects. Yields increase by 2.54 quintals per acre (0.64 tons per hectare) without control variables and rise to 2.83 quintals per acre (0.70 tons per hectare) with controls, both statistically significant at the 5% level. Similarly, revenue from wheat increases by 5,150.18 INR per acre (12,731.69 INR per hectare) without controls and 5,712.28 INR per acre (14,118.45 INR per hectare) with controls, achieving statistical significance at the 1% and 5% levels, respectively. These results underscore the substantial impact of the cut-soiler treatment on wheat, even after accounting for potential confounding variables.

Table 3 shows the varied impacts of the cut-soiler treatment on wheat yields over different years, highlighting the temporal variations in agronomic responses. For the plots treated in 2020, the yields show a robust increase of 2.61 quintals per acre (0.65 tons per hectare) without control variables, a statistically significant result at the 1% level. When control variables are incorporated, the yield increment slightly decreases to 2.37 quintals per acre (0.59 tons per hectare), statistically significant at the 1% level, indicating the consistent positive effects of the treatment. By contrast, the plots treated in 2022 exhibit less consistent outcomes. Without control variables, the yield increase is a modest 0.84 quintals per acre (0.21 tons per hectare), and even with control variables, where a higher growth of 2.91 quintals per acre (0.72 tons per hectare) is observed, neither result achieves statistical significance. Moreover, the economic impact mirrors the agronomic results. The revenue from the 2020-treated plots shows a significant increase from 5,366 INR to 6,824 INR per acre (13,254 INR to 16,857 per hectare), as statistically significant at the 1% level. Conversely, the revenue enhancements for the plots treated in 2022 fail to meet the statistically significant thresholds, further underscoring the inconsistent economic returns from the treatment in that year.

**Table 3:** Results from the heterogeneous DID analysis: Wheat cultivation.

	Yield (quintals/acre)				Revenue (INR/acre)			
	2020		2022		2020		2022	
	without X	with X	without X	with X	without X	with X	without X	with X
ATET	2.61^***^	3.37^***^	0.84	2.91	5366.27^***^	6824.01^***^	1954.25	6206.89
	(0.43)	(1.24)	(0.86)	(3.59)	(803.23)	(2402.21)	(1743.22)	(7396.99)
*N*	320	320	320	320	320	320	320	320
Cluster	64	64	64	64	64	64	64	64

Note: ATET, Average Treatment Effect on the Treated. The estimator used for the models is regression adjustment. In addition to the variables in the table above, the models are adjusted for a range of covariates (X), including household-level variables such as the gender, age, and education level of the household head, along with the number of family members engaged in farm labor. Additionally, plot-level characteristics are accounted for, including plot size, the use of tube well irrigation, the use of gypsum, the household head’s perception of salinity, and the application volumes of inputs such as UREA, DAP, ZINC, MOP, and organic fertilizers. Standard errors adjusted for 64 clusters are shown in parentheses.

***p<0.01,

**p<0.05,

*p<0.1.

**Fig 5:**
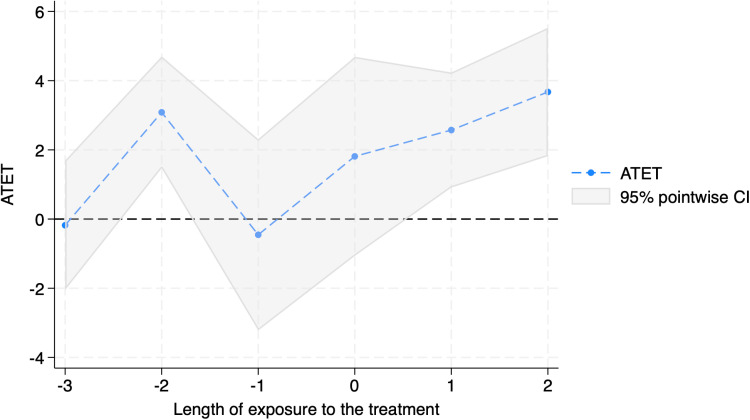
Dynamic Treatment Effects of Cut-soiler Implementation on Wheat Yields.

**Fig 6:**
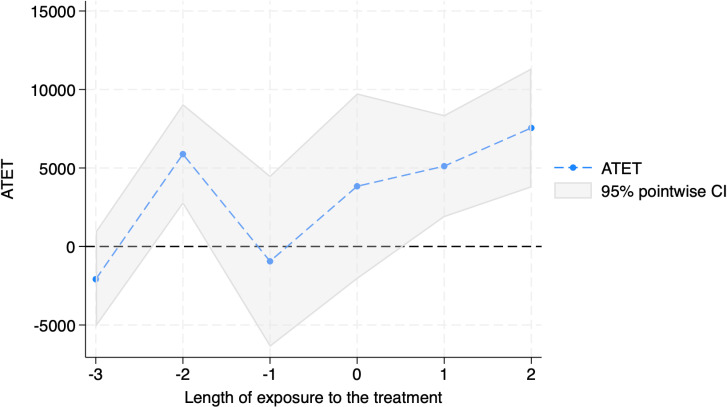
Dynamic Treatment Effects of Cut-soiler Implementation on Wheat Revenues.

The above findings are shown in Table 4 and Figs 5 and 6. Table 4 suggests that the effects of the cut-soiler treatment may become more pronounced over time, with more evident benefits two years after treatment compared to the immediate year following application. Fig 5 corroborates this result, showing no significant increase in yield in the year immediately following treatment but a substantial surge in the second year. Similarly, as depicted in Fig 6, revenue trends align with these yield increases, indicating that the v benefits from the treatment accrue more significantly with time.

**Table 4:** Results from the dynamic treatment effects estimation: Wheat cultivation.

	Yield (100kg/acre)		Revenue (INR/acre)	
Exposure	without X	with X	without X	with X
0 (Treatment year)	1.12^**^	2.47	2715.15^***^	5116.71
	(0.45)	(1.59)	(744.94)	(3227.74)
1 year after treatment	2.80^***^	3.57^**^	5607.82^***^	7105.35^**^
	(0.70)	(1.73)	(1389.83)	(3372.56)
2 years after treatment	3.73^***^	4.37^***^	7775.82^***^	8976.76^***^
	(0.54)	(0.95)	(1110.48)	(2842.63)
*N*	320	320	320	320
Cluster	64	64	64	64

Note: Besides the variables in the table above, the models are adjusted for a range of covariates (X), including household-level variables such as the gender, age, and education level of the household head, along with the number of family members engaged in farm labor. Standard errors adjusted for 64 clusters are shown in parentheses.

***p<0.01,

**p<0.05,

*p<0.1.

### Robustness check: analysis of covariance

Table 5 presents the impact of the cut-soiler treatment on rice plots, as evaluated through ANCOVA. The yield results align closely with those in Table 2, with significant increases observed. Specifically, the treated plots show a yield increase of 2.15 quintals per acre (0.54 tons per hectare) without controls, rising to 2.59 quintals per acre (0.64 tons per hectare) with controls, both statistically significant at the 1% and 5% levels, respectively. However, while revenue outcomes mirror those in Table 2, they fail to reach statistical significance. This suggests that while yield improvements are evident, translating these into significant economic benefits may depend on other factors, such as market conditions and input costs.

**Table 5:** Results from the ANCOVA: Rice cultivation.

	Yield (100kg/acre)		Revenue (INR/acre)	
	without X	with X	without X	with X
Treated	2.15^**^	2.59^***^	-3,434.58	-2015.99
	(1.06)	(0.79)	(8,699.05)	(11,193.34)
Pre-treatment outcome	0.90^***^	0.87^***^	1.01^***^	1.03^***^
	(0.07)	(0.10)	(0.11)	(0.11)
Plot size (acre)		0.11^*^		-406.82^*^
		(0.06)		(210.00)
Tube well irrigation (1=yes)		0.06		7,455.62^*^
		(1.38)		(4,241.60)
HH’s perception of salinity		0.03		178.98
		(0.25)		(828.94)
UREA applied (kg/acre)		2.04^***^		2,288.71^**^
		(0.75)		(971.97)
DAP applied (kg/acre)		-0.90		165.83
		(1.27)		(8,996.24)
ZINC applied (kg/acre)		-5.92^**^		-9,230.28^***^
		(2.27)		(2,138.75)
MOP applied (kg/acre)		0.06		2,599.77
		(0.33)		(2,134.75)
Organic fertilizer applied (kg/acre)		-0.00		-6.92
		(0.00)		(16.48)
Gypsum applied (1=yes)		-0.22		-4,313.59
		(0.79)		(3,217.32)
Constant	1.04	-1,374.21^***^	-2,382.82	310.37
	(0.96)	(423.65)	(3,230.19)	(8,180.17)
*N*	320	320	320	320
R^2^	0.58	0.61	0.53	0.58
Year FE	YES	YES	YES	YES

Note: Besides the variables in the table above, the models are adjusted for a range of covariates (X), including household-level variables such as the gender, age, and education level of the household head, along with the number of family members engaged in farm labor. Standard errors adjusted for 64 clusters are shown in parentheses.

***p<0.01,

**p<0.05,

*p<0.1.

Table 6 details the effects of the cut-soiler treatment on wheat plots, also analyzed through ANCOVA. The results of wheat treated in 2020 show robust yield improvements: 2.06 quintals per acre (0.52 tons per hectare) without controls and 2.25 quintals per acre (0.56 tons per hectare) with controls, both statistically significant at the 1% level. Similarly, wheat treated in 2022 shows even higher increases, with yields rising to 3.50 quintals per acre (0.87 tons per hectare) without controls and 4.19 quintals per acre (1.05 tons per hectare) with controls, both statistically significant at the 1% level. This consistency reinforces the effectiveness of the cut-soiler treatment in enhancing wheat productivity.

**Table 6:** Results from the ANCOVA: Wheat cultivation yield.

	Yield (100kg/acre)			
	2020		2022	
	without X	with X	without X	with X
Treated	2.06^***^	2.25^***^	3.50^***^	4.19^***^
	(0.33)	(0.48)	(0.72)	(0.71)
Pre-intervention outcome	0.60^***^	0.63^***^	0.83^***^	0.83^***^
	(0.05)	(0.06)	(0.06)	(0.07)
Plot size (acre)		0.03		0.04^*^
		(0.03)		(0.02)
Tube well irrigation (1=yes)		0.00		-0.75^***^
		(0.38)		(0.27)
HH’s perception of salinity		-0.03		0.01
		(0.07)		(0.08)
UREA applied (kg/acre)		-0.37^**^		-0.20^*^
		(0.14)		(0.12)
DAP applied (kg/acre)				
ZINC applied (kg/acre)		-0.93^**^		-0.53
		(0.44)		(0.40)
MOP applied (kg/acre)				
Organic fertilizer applied (kg/acre)		0.00		0.00
		(0.00)		(0.00)
Gypsum applied (1=yes)		-0.78^*^		-0.25
		(0.41)		(0.36)
Constant	4.56^***^	5.40^***^	1.35^*^	2.26^*^
	(0.70)	(1.16)	(0.76)	(1.17)
*N*	320	320	320	320
*R* ^*2*^	0.20	0.22	0.26	0.27
Year FE	YES	YES	YES	YES

Note: In addition to the variables in the table above, the models are adjusted for a range of covariates (X), including household-level variables such as the gender, age, and education level of the household head, along with the number of family members engaged in farm labor. Standard errors adjusted for 64 clusters are shown in parentheses.

***p<0.01,

**p<0.05,

*p<0.1.

Table 7 extends this analysis to wheat revenue. For wheat treated in 2020, the revenue gains are substantial: 4,353 INR per acre (10,753 INR per hectare) without controls and 4,712 INR per acre (11,648 INR per hectare) with controls, both statistically significant at the 1% level. Wheat treated in 2022 shows even higher revenue increases: 6,760 INR per acre (16,708 INR per hectare) without controls and 8,157 INR per acre (20,142 INR per hectare) with controls, also significant at the 1% level.

**Table 7:** Results from the ANCOVA: Wheat cultivation revenue.

	Revenue (INR/acre)			
	2020		2022	
	without X	with X	without X	with X
Treated	4,353.55^***^	4,712.88^***^	6,760.07^***^	8,157.36^***^
	(657.38)	(964.67)	(1,679.65)	(1,667.51)
Pre-intervention outcome	0.62^***^	0.65^***^	0.82^***^	0.83^***^
	(0.06)	(0.07)	(0.06)	(0.08)
Plot size (acre)		56.32		75.04
		(59.87)		(46.71)
Tube well irrigation (1=yes)		-44.33		-1,558.33^***^
		(760.32)		(568.15)
HH’s perception of salinity		-60.44		25.05
		(148.61)		(160.29)
UREA applied (kg/acre)		-780.21^**^		-402.53^*^
		(295.32)		(239.68)
DAP applied (kg/acre)				
ZINC applied (kg/acre)		-1,933.05^**^		-1,028.45
		(847.06)		(821.84)
MOP applied (kg/acre)				
Organic fertilizer applied (kg/acre)		1.61		2.39
		(1.82)		(2.13)
Gypsum applied (1=yes)		-1,623.85^*^		-551.29
		(829.23)		(738.63)
Constant	7,731.25^***^	9,508.38^***^	1,656.69	3,569.88
	(1,421.58)	(2,349.77)	(1,615.60)	(2,396.86)
*N*	320	320	320	320
*R* ^*2*^	0.28	0.30	0.32	0.33
Year FE	YES	YES	YES	YES

Note: Besides the variables in the table above, the models are adjusted for a range of covariates (X), including household-level variables such as the gender, age, and education level of the household head, along with the number of family members engaged in farm labor. Standard errors adjusted for 64 clusters are shown in parentheses.

***p<0.01,

**p<0.05,

*p<0.1.

These results collectively highlight the cut-soiler treatment’s consistent effectiveness in improving yields and revenues for rice and wheat plots. The findings suggest that the treatment’s efficacy is sustained across different models and becomes more pronounced when considering advanced controls. While some variability is observed in economic outcomes, particularly for rice, the overall trends underscore the transformative potential of the cut-soiler intervention in enhancing crop productivity and economic returns for farmers.

### Financial analysis of cut-soiler technology

**Fig 7:**
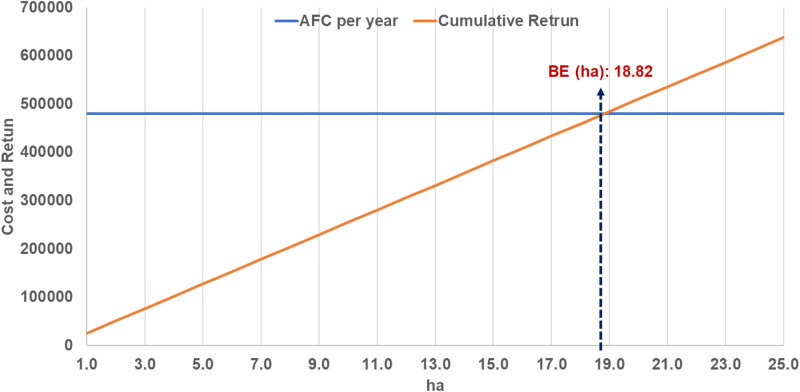
Break-Even Analysis of Annual Fixed Costs and Cumulative Returns for Cut-soiler Usage.

The estimated value of the annualized fixed cost was INR 480,255, and the annual savings from the rice-wheat system and variable cost were INR 34,262 and 8,750 per hectare, respectively. The break-even analysis results demonstrate that at least 18.82 hectares of the rice-wheat system must be managed with the cut-soiler to meet its annual fixed costs (Fig 7).

Table 8 presents the assessment of financial viability indicators, including NPV, BCR, and IRR, across various coverage scenarios. The analysis shows that covering 20 hectares with the cut-soiler yields favorable NPV (>0), BCR (>1), and IRR (>10%) values, thereby affirming the investment’s potential for reclaiming sodicity-affected areas. Additionally, the indicators suggest that as the area of effective utilization expands, the associated benefits also increase. These outcomes highlight the necessity of advocating for the adoption of cut-soiler practices through community-based clusters rather than on an individual farmer level.

**Table 8:** NPV, BCR, and IRR across varying cut-soiler coverage scenarios.

Particular	Scenarios of coverage of cut-soiler				
	20 ha	25 ha	30 ha	35 ha	40 ha
NPV (INR)	1492519	2795501	4098483	5401466	6704448
BCR	1.40	1.75	2.10	2.45	2.80
IRR (%)	16.69	22.03	27.13	32.87	36.94

Note: NPV, Net Present Value. BCR, Benefit-Cost Ratio. IRR, Internal Rate of Return.

## Discussion

The economic implications of adopting cut-soilers for rice and wheat cultivation in India were evaluated using projections based on observed yield increases and subsequent revenue enhancements. While previous studies conducted on experimental farms in regions such as Uzbekistan, India, and Tanzania [4,7,9,14–16] have consistently demonstrated the potential of cut-soilers to enhance crop productivity, this study offers a novel contribution by shifting the focus to real-world applications in farmer-managed fields.

Our findings further reinforce cut-soilers’ effectiveness in boosting agricultural yields under practical farming conditions. The analysis reveals that adopting cut-soiler technology could increase rice yields by approximately 2.12 quintals per acre (0.52 tons per hectare) and wheat yields by about 2.54 quintals per acre (0.64 tons per hectare). These results underscore the technology’s promise in semi-controlled experimental settings and in addressing the complexities of real-world farming systems.

Translating these yield enhancements into monetary terms using the projected 2022 unit prices—4,014.803 INR per quintal for rice and 2,092.803 INR per quintal for wheat—suggests potential revenue increases of 8,511.38 INR for rice farms and 5,315.72 INR for wheat farms. Adjusting for the average farm size in Haryana (2.25 hectares) and Punjab (3.66 hectares), the total projected annual revenue increases to 116,718 INR for rice and 72,909 INR for wheat farms in Haryana, and similarly to 189,999 INR for rice and 118,838 INR for wheat farms in Punjab. In addition to these economic benefits, the cut-soiler is designed with user-friendliness in mind. Its intuitive and straightforward design ensures ease of use, even for beginners, significantly lowering the barriers to adoption posed by operational complexity.

However, despite these promising projections, the high initial cost of the cut-soiler, currently sold in Japan for 6,270,000 Japanese Yen (JPY) (equivalent to 3,652,857 INR as of August 26, 2024), remains a significant barrier to widespread adoption. According to the break-even analysis, the annual fixed cost of a cut-soiler is approximately 480,255 INR, requiring at least 18.82 hectares of operation in a rice-wheat system to cover this cost. However, as the average farm sizes in Haryana and Punjab fall well below this threshold, achieving economic feasibility at the individual farm level is challenging.

Several strategies can be employed to address these financial challenges and make the technology more viable. Community group purchases are promising, as they distribute the financial burden across multiple farmers. Financial analyses show that cut-soilers become more cost-effective at larger scales. For example, managing 20 hectares results in a positive NPV, a BCR exceeding 1, and an IRR above 10%. These findings highlight the potential of community-based clusters to achieve economic efficiency and promote broader adoption compared to individual ownership.

In addition to group purchases, optimized rental models could further reduce costs. For instance, shorter rental periods better align with the cut-soiler’s ability to quickly treat an acre, avoiding inefficiencies associated with traditional month-long agreements. Establishing custom hiring centers in regions like Haryana and Punjab could simplify rental access. In contrast, peer-to-peer rental systems, modeled after existing tractor-sharing practices, could provide smallholders with more flexible and affordable options.

Government subsidies and financial assistance programs offer another pathway to offset the high initial cost. Given that cut-soilers improve productivity in salt-affected regions and reduce environmental harm, such as air pollution from rice residue burning, subsidies can be justified on both agricultural and environmental grounds.

Additionally, transitioning to mass production and relocating manufacturing facilities to lower-wage countries could significantly reduce production costs, making the technology more accessible to smallholder farmers.

Implementing these strategies can significantly improve the affordability and accessibility of cut-soiler technology, enabling wider adoption. This would enhance agricultural productivity in salt-affected regions and contribute to environmental sustainability by reducing harmful practices like rice residue burning. Together, these approaches demonstrate cut-soilers’ potential to benefit farming communities economically and environmentally.

Finally, while these findings highlight cut-soilers’ potential, their effectiveness may vary depending on agro-climatic conditions, soil properties, and other localized factors. This variability underscores the need for cautious interpretation and consideration of regional differences when generalizing results to broader agricultural contexts. Future research should prioritize multi-location trials across diverse environments to better understand the technology’s adaptability and identify region-specific factors, such as soil type, water availability, and crop management practices. Incorporating farmer-level variables, such as management skills and socio-economic conditions, into future analyses will further refine insights and support region-specific recommendations, ensuring broader applicability and adoption of cut-soiler technology.

## Conclusion

This study highlights the potential of cut-soiler technology to address root zone alkalinity, a critical challenge for agricultural productivity in salt-affected regions of the western IGP. Improving soil conditions and enhancing drainage, cut-soilers significantly increase rice and wheat yields, which is vital for India’s food security. Moreover, the technology contributes to environmental sustainability by reducing crop residue burning and its associated air pollution.

The analysis reveals that adopting cut-soiler technology can substantially increase farmers’ revenue, particularly in Haryana and Punjab. However, the large land requirements for break-even pose significant barriers to widespread adoption, particularly for smallholder farmers.

These challenges underscore the importance of financial support mechanisms, such as subsidies, optimized rental models, and community-based purchasing initiatives, to enhance affordability and accessibility.

Future research should explore the long-term impacts of cut-soiler use on soil health and productivity across diverse agro-climatic conditions. By combining technological innovation with supportive policies, the broader adoption of cut-soilers can improve farmer livelihoods, enhance agricultural sustainability, and contribute to achieving the SDGs and LDN targets.
